# Classic test and generalizability theories are both useful for cross-cultural adaptation of an allergic rhinitis health-related quality of life questionnaire

**DOI:** 10.1016/j.waojou.2021.100612

**Published:** 2021-11-29

**Authors:** Juan José Yepes-Nuñez, Edison Morales Cardenas, Carolina Gómez-García, Madelen Manco Sepúlveda, Lina Marcela Martínez, Antonio Valero, Meghan M. McConnell

**Affiliations:** a Universidad de los Andes, School of Medicine, Carrera 7 #116-5, 110111, Bogotá, Colombia; b Pulmonology Service, Internal Medicine Section, Fundación Santa Fe de Bogotá University Hospital, Carrera 7b #123-90, 110111, Bogotá, Colombia; c Unidad Alergológica, Calle 19 A #44-25, Consultorio 2405, Salud y Servicios Ciudad del Río, Medellín, Antioquia, Colombia; d Sección de Alergología, Servicio de Neumología y Alergia, Hospital Clínic de Barcelona, Universitat de Barcelona, IDIBAPS, CIBER de Enfermedades Respiratorias (CIBERES), Barcelona, Spain; e Department of Innovation in Medical Education, University of Ottawa, Ottawa, Ontario, Canada; f Department of Anesthesiology and Pain Medicine, University of Ottawa, Ottawa, Ontario, Canada

**Keywords:** Quality of life, Allergic rhinitis, Validation studies, Surveys and questionnaires

## Abstract

**Background:**

An instrument to assess Allergic Rhinitis (AR) Health-Related Quality of Life (HRQL) in adult patients was developed in Spain. No validated instrument is currently cross-culturally adapted for use in daily practice to assess HRQL in AR patients in Colombia.

**Purpose:**

The aim of this study was to evaluate the measurement performance of an AR-HRQL specific questionnaire, ESPRINT-15 (Cuestionario ESPañol de Calidad de Vida en RINiTis), in Colombian adult patients with AR using the Classic Test Theory (CTT) and the Generalizability theory (G-theory) frameworks.

**Methods:**

We conducted the cross-cultural adaptation in 2 stages. In stage 1, we evaluated comprehensibility, acceptability, and feasibility of ESPRINT-15 in healthy adults and adult patients with AR. In stage 2, we examined both reliability and validity of ESPRINT-15 scores using CTT and overall reliability applying the G-theory in adult patients with AR.

**Results:**

For feasibility and acceptability, all items showed a higher than 95% level of understanding, and modifications in the original questionnaire were unnecessary. Reliability and validity using CTT showed a high internal consistency (Cronbach's alpha and Mc McDonald's omega = 0.95) and test-retest reliability (scores from 0.70 to 0.76). The overall reliability score using G-theory was 0.75, and G-coefficients scores associated with internal consistency and test-retest reliability measures were 0.96 and 0.61, respectively. Validity using exploratory factor analysis (EFA) identified 2 factors instead of the original ESPRINT-15 4 domains. However, confirmatory factor analysis (CFA) showed good fit regarding the original model.

**Conclusions:**

The proposed cross-cultural adaptation of ESPRINT-15 showed good reliability and validity measures. Additionally, it was easy to use and administer. ESPRINT-15 can be used clinically and for research in Colombian adults' patients with AR. CTT and the G-theory can be used in epidemiological studies to adapt AR-HRQL questionnaires cross-culturally in adult patients with AR.

## Background

The high prevalence of allergic rhinitis (AR) and its effects on health-related quality of life (HRQL) have positioned this disease as one of the main chronic respiratory diseases worldwide.^1^ Globally, the prevalence of AR is around 400 million people and AR symptoms are not well recognized by patients;^2^ in Colombia, the prevalence of AR symptoms ranges from 29.5% to 33.9% for the entire population, and 29.2%–32.4% in the pediatric population.^3^^,^^4^ AR is associated with impaired social life, sleep, school, and work^5^ and several studies have explored how AR may negatively affect sleep, concentration, performance at work, learning and school activities, social life, sexuality, and sports.^6^, ^7^, ^8^, ^9^, ^10^, ^11^, ^12^

HRQL is a critical patient-important outcome measurement that describes how individuals and groups perceive physical and mental health over time.^13^ Moreover, HRQL data allows clinicians and patients to recognize and monitor the impact of diseases and might improve patients' commitment to disease management.^14^, ^15^, ^16^ AR's significant impact on HRQL and the cost of HRQL costs suggests an important public health burden.^17^^,^^18^ For instance, AR has a significant economic burden on society; in the United States, the estimated annual cost of AR is about US$2-5 billion; and in Latin-American countries, intranasal corticosteroid treatment can cost at least US$230 per annum per patient.^19^^,^^20^ In consequence, the World Allergy Organization (WAO) and the Allergic Rhinitis and its Impact on Asthma (ARIA) clinical practice guidelines recommend classifying the severity of AR based on HRQL measures.^21^, ^22^, ^23^

While multiple specific AR-HRQL questionnaires exist, most of them are heavily influenced by the cultural characteristics. In consequence, it is critical to validate and adapt their use to new populations.^24^, ^25^, ^26^ Cross-cultural adaptation of HRQL questionnaires is less expensive and less time-consuming than developing a new tool. It allows the use of HRQL questionnaires adapted in cross-national research. Such adaptation should be made using reliable methods that measure the new version's psychometric characteristics.^27^^,^^28^

The application of an instrument in another target population, another language, or another form of administration is considered a new situation. A well-known type of validation is cross-cultural validation, ie, validation when an instrument is applied in countries with different cultures and languages.^29^ Classic Test Theory (CTT) and the Generalizability theory (G-theory) have similarities and use measurement procedures to yield reliable data.^30^ Particularly, G theory enables researchers to quantify and identify the sources of inconsistencies in observed scores that arise, or could arise, over replications of a measurement procedure.^31^ Although both frameworks assess psychometric properties of HRQL questionnaires, their similarities in terms of reliability measures have never been compared in a cross-cultural adaptation study.

Heine et al^32^ described that some individual functional psychological characteristics vary across different people and societies. For example, the population of Western, Educated, Industrialized, Rich, and Democratic (WEIRD) societies exhibits different psychological characteristics when compared to the population of non-WEIRD societies. These differences have taken place in domains such as social decision making (fairness, cooperation, and punishment), independent versus interdependent self-concepts (and associated motivations), analytic versus holistic reasoning, and moral reasoning.^32^

Two generic HRQL measurement questionnaires have been translated into Spanish and cross-culturally adapted to Colombia.^33^^,^^34^ Regarding AR, a variety of specific HRQL questionnaires exist, but none have been developed or adapted to measure HRQL in Colombian adults with AR. ESPRINT-15 (Cuestionario ESPañol de Calidad de Vida en RINiTis), designed in Spain, is a validated questionnaire developed to measure HRQL in Spanish adults with AR.^35^ Scores from ESPRINT-15 are associated with high internal consistency (Cronbach's alpha: 0.92) and reproducibility (intraclass correlation coefficient: 0.80).^13^ ESPRINT-15 has also demonstrated moderate to strong correlations to symptom score questionnaires such as visual scale analog and generic HRQL questionnaires.^36^ Such findings support the applicability of ESPRINT-15 as a tool that can provide reliable and valid HRQL measurements in Colombian adults with AR.

In this article, considering that the Colombian population with AR has different cultural values than the Spanish population with the same disease, we conducted a cross-cultural adaptation process of the ESPRINT-15 in Colombian adults (over 18-years old) with AR using the using classic test theory and g-theory. We analyzed its psychometric measures such as comprehensibility, feasibility, acceptability, reliability, and validity.

## Methods

From each participant, we obtained demographic data, place of residence, duration of AR, and severity of AR according to the ARIA criteria.^37^ We assessed asthma and its severity in each participant based on the Global Strategy for Asthma Management and Prevention (GINA) criteria.^38^ The Total 4 Symptom Score (T4SS) was calculated as the sum of the 4 main nasal symptoms of rhinitis: nasal congestion, rhinorrhea, nasal itching, and sneezing. Symptoms were scored from 0 to 3 (0, no symptoms; 1, mild; 2, moderate; 3, severe), resulting in a T4SS ranging from 0 to 12. Increasing values are associated with increasing severity of disease.

### Overall study design

ESPRINT-15 is a questionnaire that includes 15 items distributed in the following domains: symptoms (5 items), daily activities (3 items), sleep (3 items), psychological impact (3 items), and overall score (1 item). The questionnaire provides domain scores ranging from 0 to 6 and an overall score ranging from 0 to 5.8. Lower scores indicate a better quality of life. The measurement goal of ESPRINT-15 is both discrimination and evaluation.^39^

The ESPRINT-15 questionnaire that we used was provided by the original developer, the Spanish Society of Allergy and Clinical Immunology. In order to get a conceptual equivalence, the original Spanish version was reviewed before it was implemented in this study.

**Fig. 1:**
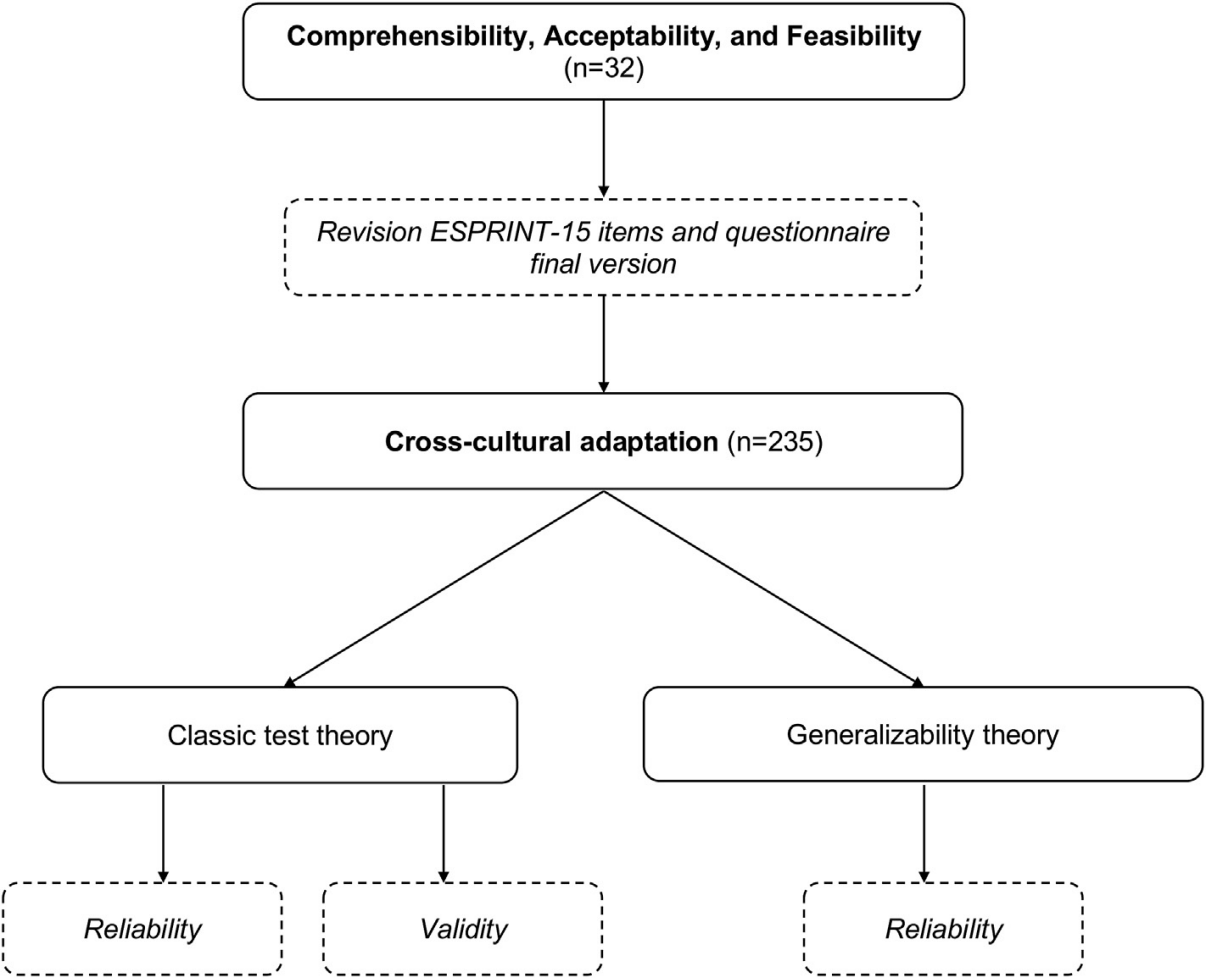
Development process of the cross-cultural adaptation for the ESPRINT-15 questionnaire in Colombia

We carried out a cross-cultural adaptation in 2 stages. Stage 1 examined the comprehensibility, acceptability, and feasibility of the ESPRINT-15 questionnaire. Stage 2 explored its reliability and validity of ESPRINT-15. All patients signed an informed consent before their inclusion in the study. Fig. 1 shows the steps developed during the cross-cultural adaptation.

### Stage 1: Comprehensibility, acceptability, and feasibility

Stage 1 was carried out by 2 researchers (CGG, JJYN) fluent in Spanish and knowledgeable in allergic diseases and HRQL.

#### Procedure

We examined comprehensibility in 2 pilot tests at this stage. In the first pilot test, we wanted to review any preliminary comprehensibility problems with the questionnaire, and for this purpose we asked twelve adults who did not suffer from the disease to fill out the questionnaire. After reviewing their comments, we conducted a second pilot test including 20 adults with AR to judge comprehensibility, relevance, and completeness of the ESPRINT-15 questionnaire.^29^ We conducted the pilot tests in an Allergy Unit located in a private health care institution in Medellín, Colombia.

In both pilot tests, we included 3 questions after each item. First, patients answered yes or no to the question “*Did you understand the item?”*. The other 2 questions were open-ended: *“What did you not understand?”* and *“What suggestions do you have to change it?”*. Upon questionnaire completion, a verbal pre-testing was carried out by asking the patients to comment on the questionnaire items and their perceived meaning. We reviewed the answered questionnaires for problematic items, responses, statements, and words in both stages.

We assessed acceptability by asking the same 32 respondents whether they were willing to fill out the questionnaire by themselves. Feasibility was evaluated by measuring the completion time of ESPRINT-15.

### Stage 2: Reliability and validity

Five co-authors (MM, LM, EM, CG, JYN) were trained to collect information from interviews, specifically clinical characteristics of the disease, but they did not interfere with questionnaire completion.

#### Procedure

We conducted the second stage at the same health care institution where stage 1 was developed. We enrolled new adult patients with AR when they attended the health care institution to start allergenic immunotherapy treatment. Patients were assessed twice with a 4-week interval between visits. This interval was chosen according to a time interval applied in a previous study to assess test-retest reproducibility and sensitivity to change of ESPRINT-15.^39^ For test-retest reproducibility, we considered that patients did not change their clinical condition between both visits because onset of clinical efficacy occurs within 2–4 months.^40^

At both visits, patients were invited to fill in a hard copy version of the ESPRINT-15 questionnaire. All returned questionnaires were examined for missing answers using a consistent protocol performed by the 5 co-authors. Patients were telephoned, up to 3 times, to resolve ambiguous responses. If it was not possible to clarify the answers, the data were entered as ‘0ʼ in the database, resulting in participant exclusion.

### Sample size estimation

We did not calculate a sample size calculation for stage 1. In stage 2, we applied a method developed to calculate the approximate number of patients required to obtain an exact confidence interval of the desired width for a certain type of intraclass correlation in one-way and two-way analysis of variance (ANOVA) model.^41^ Setting the level of significance at *α* = 0.05, for intraclass correlation 0.7 ($\tilde{p}I$=0.7), with a width of 0.2 (*ω* = 0.2) and if each patient is measured by the same set of *κ* questionnaires twice (*κ* = 2) which are the only questionnaires of interest, 104 patients would be required (η = 104). According to the authors of the model, the sample size approximation is less least accurate for *κ* = 2 and $\tilde{p}I$≥ 0.7 and its accuracy can be improved by adding 5 $\tilde{p}I$ to η in special cases. Therefore, we estimated a final sample size of 109 patients (η = 109). We applied a convenience sampling to include healthy Colombian adults and adult patients with AR in both stages.

### Statistical analysis

Statistical analyses were performed using IBM SPSS 27 for Mac (IBM, Corp, Armonk, NY) while confirmatory facto analysis was performed using an IBM SPSS Amos 26 (IBM, Corp, Armonk, NY). To calculate generalizability coefficients, we utilized G-String IV software (available at https://healthsci.mcmaster.ca/merit/research/g_string_v). We used descriptive statistics of mean, standard deviation (SD), frequencies, and percentages to summarize the data.

#### Comprehensibility

We assessed comprehensibility in stage 1 to ensure that the original meaning of ESPRINT-15 questionnaire was not lost or altered while reaching cultural equivalence.^29^ We calculated an average number of “yes” responses for each ESPRINT item to the question, *“Did you understand the previous item?”.* We established a threshold of 95% to accept comprehensibility for each item.

#### Acceptability and feasibility

Acceptability refers to the question of whether or not patients were willing to do something, and feasibility refers to whether or not they were able to do it.^29^ To evaluate acceptability, we assessed completeness of data in stage with a criterion for missing data <10%. Feasibility was assessed by calculating the time patients took to complete the questionnaire both in stage 1 and stage 2.

#### Reliability

Broadly conceived, reliability provides measures of consistency of questionnaire scores. The reliability of ESPRINT-15 was assessed in stage 2 using both CTT and G-theory.

*Classical Test Theory* (CTT) evaluates reliability by measuring *internal consistency* and *reproducibility* of scores. *Internal consistency* refers to the consistency among items on the questionnaire, while *reproducibility* (the test-retest reliability) establishes the stability of the questionnaire over time in a stable population.^42^ In the present study, we assessed internal consistency by Cronbach's alpha and McDonald's Omega coefficients for each domain as well as the overall test. McDonald's Omega is considered one the best alternatives to calculate reliability.^43^^,^^44^ A Cronbach's alpha value between 0.7 and 0.9 was considered enough to achieve a proper level of internal consistency.^45^ *Reproducibility* was evaluated using intra-class correlations (ICC). To assess the internal consistency of ESPRINT-15 scores, we took the average score between the first and second measurements.

*Generalizability Theory*. In addition to using CTT, we used G-theory^46^^,^^47^ to determine the overall reliability of ESPRINT-15, as well as internal consistency of ratings. G-theory allows for the estimation of multiple sources of variance, and therefore provides a useful framework for estimating the reliability of various types of assessments. In the present study design, individual items (i) are nested in a survey domain (i:d) and are crossed with time (t) and patients (p).

#### Validity

Validity is the degree to which the questionnaire measures what it is supposed to measure.^42^ In stage 2, we conducted exploratory factor analysis (EFA). We applied the Bartlett's Sphericity Test and Kaiser–Meyer–Olkin (KMO) Test to determine if the data was suitable to perform an EFA. The extraction of the axes for the factor analysis was based on the choice of eigenvalues greater than 1 and parallel analyses were used to confirm how many factors to retain. A rotation of the axes was be carried out to obtain greater representativeness in terms of factor loads (rotation promax, varimax or quartimax). The items that sufficiently saturated the retained factors were selected, setting a limit of 0.30 or higher for the selection. The analysis was completed with a scree plot. We conducted a confirmatory factor analysis (CFA) via Structural Equation Modelling (SEM) to test the questionnaire's structure. We used statistical tests to determine and compare goodness of fit of the models in absolute fit indices including Chi-squared test, χ2/df ratio, and standardized root mean square residual (SRMR). We used for relative fit indices, the Tucker-Lewis index (TLI), and indices of fit like the root mean square error of approximation (RMSEA), Bentler's Comparative Fit index (CFI), and the coefficient of determination (CD). RMSEA values lower than 0.05 indicate a good fit to the data, values between 0.05 and 0.08 an acceptable fit, values between 0.08 and 0.10 a marginal fit, and values greater than 0.10 a poor fit.^48^ For the CFI, NFI, and TLI, values greater than 0.90 indicate a good fit to the data.^49^ To extract the variances from the data and to examine the factor structure, we used robust maximum likelihood estimates (MLR).^50^

We analyzed the reliability and validity of ESPRINT-15 scores using the first 14 items of the ESPRINT-15 questionnaire. We excluded the last item from the analyses because it represents an overall state of health and the measurement scale for retrieving the score was different from that used in the other 14 items. An English version of the ESPRINT-15 questionnaire is shown in Supplement 1.

##### Concurrent validity

We assessed concurrent validity using Spearman's rho coefficient to establish the correlation between the first 14 items of the ESPRINT-15 questionnaire and the overall score (item 15).

##### Discriminatory ability

We performed a Kruskal-Wallis test to evaluate the discriminant validity of the questionnaire and to compare the different median values obtained from patients with different severity of AR. Finally, a Mann Whitney *U* test was also conducted to explore if the ESPRINT-15 domains values can discriminate between patients with AR and history of asthma compared with patients with AR without having asthma.

## Results

### Baseline socio-demographic and clinical characteristics

Two hundred sixty-seven patients were included in the study; 32 patients were enrolled in stage 1 and 235 adult patients with AR in stage 2. Table 1 describes the characteristics of the patients including the classification of the severity of AR, the T4SS values, and the presence of asthma as comorbidity. In stage 1, the median age of patients was 32 (IQR, 22.75–43) and the majority were female (n = 21, 65.59%). Moderate/severe intermittent and persistent AR was prevalent in this sample (n = 5, 75% in each classification). All patients completed the questionnaire. In stage 2, the questionnaire was administered to 265 patients. Forty patients were excluded from the analysis due to inadequate questionnaire completion. In total, data from 235 patients who answered 2 times the ESPRINT-15 questionnaire without missing values was selected for this stage analysis. Most patients at this stage were females (n = 181, 77%), their median age was 28 (IQR, 22–36) with the majority living in an urban area (n = 181, 77.02%). The most common type of AR classification was persistent moderate/severe (n = 191, 81.28%) and most patients reported they had asthma under control (n = 84, 71.18%). In stages 1 and 2, the median T4SS values reported by patients were 5^3^, ^4^, ^5^, ^6^, ^7^, ^8^ and 6 (4–9), respectively.

**Table 1:** Socio-demographic and clinical characteristics of Colombian adults with allergic rhinitis.

	Comprehensibility. Acceptability, and Feasibility (phase 1)	Reliability and Validity (phase 2)
**Sample size, n**	32	235
**Age, years, Me (IQR)**	32 (22.75–43)	28 (22–36)
**Gender, male: female, n, (%)**	11 (34.41), 21 (65.59)	54 (23), 181 (77)
**Classical AR classification n (%)**^a^		
Perennial	20 (100) a	231 (98.30)
Seasonal	0 (0) a	4 (1.70)
**ARIA AR classification, n (%)**^a^		
Mild Intermittent	5 (25) a	19 (8.09)
Moderate/severe Intermittent	8 (40) a	10 (4.26)
Mild Persistent	2 (10) a	15 (6.38)
Moderate/severe Persistent	5 (25) a	191 (81.28)
**GINA asthma symptoms, n (%)**		
Yes	20 (100) a	118 (50.21)
No	0 (0)	117 (49.79)
**GINA asthma classification, n (%)**		
Well controlled	20 (100) a	84 (71.18)
Partly controlled	0 (0) a	33 (27.96)
Uncontrolled	0 (0) a	1 (0.86)
**Nasal symptoms present in last 2 weeks, n (%)**		
Sneezing	2 (1–2) a	2 (1–2)
Rhinorrhea	1 (1–2) a	2 (1–2)
Nasal pruritus	2 (1–2) a	2 (1–3)
Nasal congestion	1 (0–2) a	2 (1–3)
Olfactory loss	0.5 (0–2) a	1 (0–3)
**Ocular symptoms present in last 2 weeks, n (%)**		
Ocular pruritus	2 (1–3) a	2 (1–3)
Watery eyes	1 (0–2) a	1 (0–2)
Red eyes	1 (0–2) a	2 (1–3)
**T4SS, Me (IQR)**	5 (3–8) a	6 (4–9)
**Habitation, n (%)**		
Urban area	32 (100)	181 (77.02)
Suburban area	0 (0)	38 (16.17)
Rural area	0 (0)	9 (3.83)
No reported	0 (0)	7 (2.98)
**Education level, n (%)**		
Primary school	3 (9.38)	4 (1.70)
Secondary school	12 (37.50)	24 (10.21)
Technical institute	7 (21.88)	81 (3447)
University	10 (31.25)	126 (53.62)

AR: Allergic Rhinitis; GINA: Global Strategy for Asthma Management and Prevention; Me: Median; IQR: Interquartile Rank; SD: Standard Deviation; T4SS: Total 4 Symptoms Score.

a Data reported for 20 patients with allergic rhinitis

### Comprehensibility

The comprehensibility of ESPRINT-15 was easy as there were no problems with its understanding. Respondents found minor issues in items 3, 4, and 7 due to these items included some words that are culturally used in Spain. However, all items showed a higher than 95% level of comprehensibility in all respondents; therefore, we did not modify any items of the questionnaire. In general, respondents described that the open-ended questions were understandable and did not provide additional feedback to improve their understandability. The original items of the complete results of the pilot comprehension test are presented in Supplement 2.

### Acceptability

Both healthy adults and patients with AR were instructed to fill out in the questionnaire. There were not missing data for all items of ESPRINT-15.

### Feasibility

Overall, the questionnaire was well understood. Median time to completion was 6.5 (IQR, 4–10) minutes in stage 1, and 10 (IQR, 7–10) minutes in stage 2. Respondents did not report issues regarding the time spent to complete the ESPRINT-15 questionnaire.

### Reliability

#### Classical test theory

##### Internal consistency

ESPRINT-15 achieved good internal consistency and acceptable test-retest reliability applying statistical methods of the classical test theory. Item correlations ranged from 0.49 to 0.83. The lower item correlation was reported for the overall score item (item 15). The overall consistency of the instrument was 0.95 for Cronbach's alpha and for McDonald's Omega. When each item was removed, the values of the alpha coefficient ranged between 0.94 and 0.95. In the case of the Omega coefficients, all coefficients reported lower values than the Cronbach's alpha ones with a range between 0.89 and 0.91. The removal of an item from the questionnaire did not reduce the level of overall consistency of the instrument (Table 2).

**Table 2:** Cronbach's alpha and McDonald's Omega values the ESPRINT-15 questionnaire

Items	Item scale correlation	Cronbach' s alpha (one item removed)	McDonald's Omega *(one item removed)*
Item 1	0.58	0.95	0,91
Item 2	0.69	0.95	0,90
Item 3	0.78	0.95	0,90
Item 4	0.55	0.95	0,91
Item 5	0.71	0.95	0,90
Item 6	0.82	0.95	0,90
Item 7	0.85	0.94	0,89
Item 8	0.85	0.94	0,89
Item 9	0.80	0.95	0,90
Item 10	0.70	0.95	0,90
Item 11	0.82	0.95	0,90
Item 12	0.78	0.95	0,90
Item 13	0.75	0.95	0,90
Item 14	0.83	0.95	0,89
Item 15	0.49	0.95	0,90

##### Reproducibility (test-retest reliability)

Two hundred thirty-five patients answered the questionnaire twice, with an interval of approximately 4 weeks. ICC achieved values ranged from 0.70 (95% CI 0.61–0.77) (symptoms domain) to 0.76 (95% CI 0.69–0.82) (psychological affectation domain) for individual domains scores (Table 3).

**Table 3:** Intraclass correlation coefficient.

ESPRINT 15 domains	ICC	95% CI
Symptoms	0.70	0.61–0.77
Daily activities	0.71	0.63–0.78
Sleeping	0.74	0.66–0.80
Psychological affectation	0.76	0.69–0.82

ICC: intraclass correlation coefficient. CI: confidence interval

### G-theory findings

G-theory was used to estimate the amount of variance associated with different sources of error. Variance components were estimated for patients (p; n = 235), time (t; n = 2), domain (d; n = 4: symptoms, daily activities, sleeping, and psychological affection) and item (i; n = 14) nested in domain (i:d). The majority of variance (68%) was attributed to individual differences across patients, indicating that ESPRINT-15 was able to reliably differentiates between patients. Interaction between patient and time accounted for nearly 20% of variability in ESPRIT-15 scores, suggesting that differences between the two time periods varied depending on the patient. A small amount of variance was attributed to patients by domain interaction suggesting that domain scores varied across patients. A small amount of variance was attributed to other facets (<2%) and interactions between facets (<3%), suggesting that ESPRINT-15 scores did not vary substantially across time, or items nested in domains. Overall, only 1.6% of variance was attribute to random error. Table 4 displays the facets and variance components for ESPRINT-15.

**Table 4:** Generalizability theory.

Factors	Variance component	% Variance
Patient (p)	1.04	68.22%
Time (t)	0.01	0.86%
Domain (d)	0.03	2.17%
Item (i); d	0.01	0.60%
pt	0.30	19.82%
pd	0.0	3.16%
pi:d	0.03	1.84%
td	0.00	0.70%
ti:d	0.00	0.00%
ptd	0.03	1.78%
pti:d	0.02	1.58%

Overall Generalizability Coefficient: 0.75

Variance components were then used to calculate generalizability coefficients (G-coefficients). The overall reliability of ESPRINT-15 was 0.75, which suggests that ESPRINT-15 can generate highly reliable scores that can be used to differentiate between patients based on the AR-HRQL measure. We also calculated G-coefficients that correspond to measures of test-retest reliability (ie, average correlation between time 1 and time 2) and measures of internal consistency (ie, Cronbach's Alpha, or the average correlation between the 14 items: domains) by treating time or item: domain variance components as random facets. The G-coefficient associated with test-rest reliability was 0.61, while the G-coefficient associated with internal consistency of ESPRINT-15 scores was 0.96.

### Validity

The first 14 items of ESPRINT-15 and their corresponding likely domains were considered for an EFA. Bartlett's sphericity test (χ^2^ 3138.96, p < 0.000) indicated a correlation between the items of the questionnaire. Also, the KMO measurement of sampling adequacy (0.93) showed that data was suitable for the EFA. Initially, the EFA was conducted using principal axis factoring without rotation. Considering the eigenvalues' criterion (eigenvalues above 1), two factors were extracted (eigenvalues of 8.80, and 1.04), and the cumulative variance explained was 70.36%. Hence, an oblique (promax) rotation was performed. All factors were higher than 0.3 for all domains, suggesting that the item distribution and the domain framework would change in contrast to the original structure. Factor 1 included 8 items, ie, from items 1 to 5, and items 9 to 11, and they were entered with item loadings ranging from 0.87 to 0.51. This factor was designated physical function. Six items (items 6 to 8 and items 12 to 14) were entered for factor 2, which was designated as psychological function. Table 5 shows the rotated factor matrix obtained with factor loading of items, and supplement 3 displays the scree plot.

**Table 5:** Exploratory factor analysis.

	Factor^a^		Original domains ESPRINT-15
	1	2	
Item 10	0.87	–	Sleeping
Item 11	0.75	–	
Item 9	0.72	–	
Item 3	0.70	–	Symptoms
Item 5	0.68	–	
Item 4	0.65	–	
Item 1	0.62	–	
Item 2	0.51	–	
Item 14	–	0.98	Psychological affectation
Item 12	–	0.98	
Item 13	–	0.87	
Item 6	–	0.61	Daily Activities
Item 8	0.37	0.57	
Item 7	0.43	0.49	

Extraction Method: Principal Axis Factoring. Rotation Method: Promax with Kaiser Normalization.

a Rotation converged in 3 iterations

**Fig. 2:**
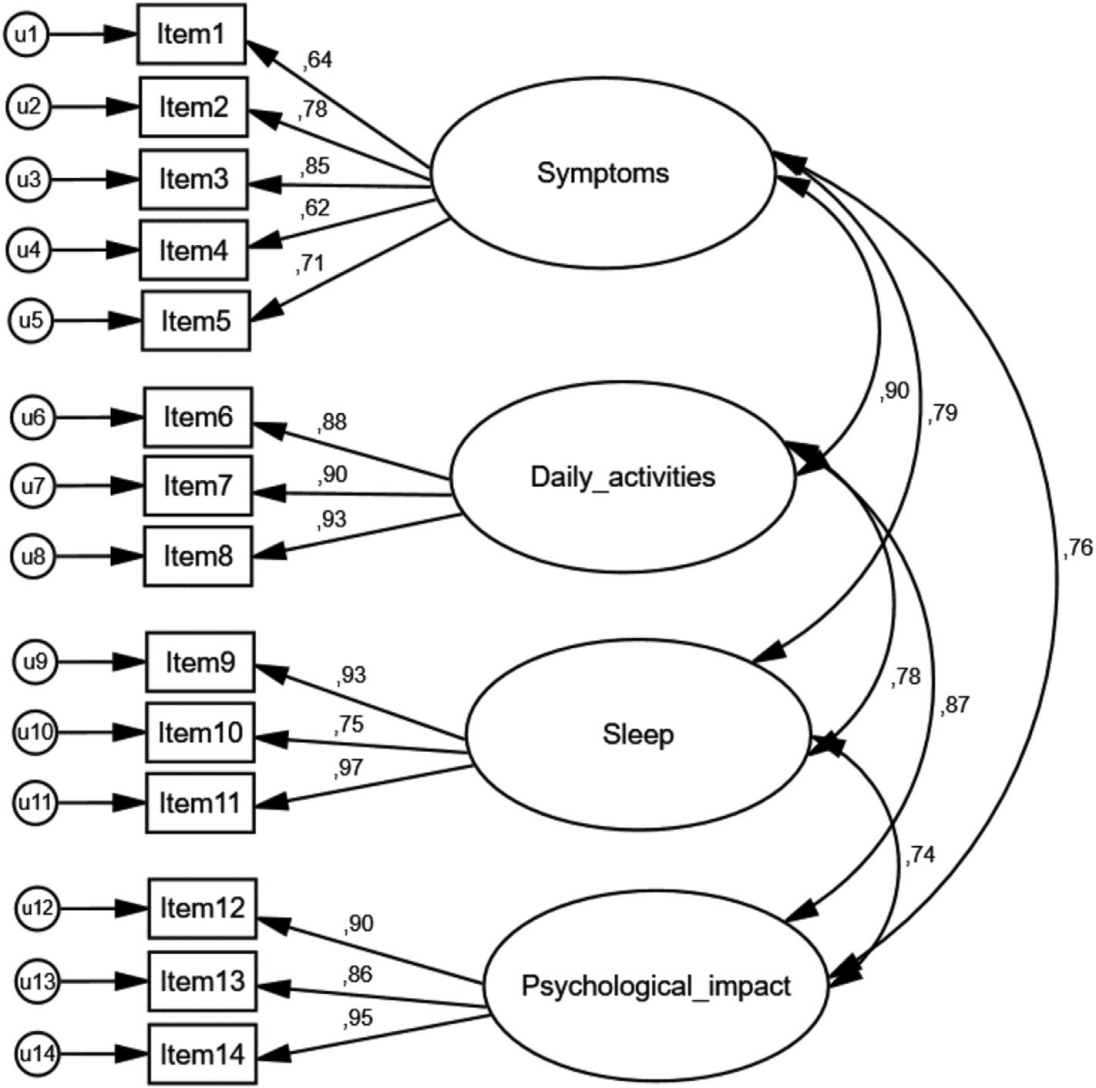
Factor structure of ESPRINT-15 in Colombian adults with AR. Items are represented as rectangles, latent variables (factors) as ellipses and loading onto factor loadings as arrows. The error terms for the observable variables are shown as a circle plus arrow for each factor. The model showed the original four-factor structure of the ESPRINT-15

To obtain an adequate model for ESPRINT-15 in Colombia, CFA with MLR estimation was conducted. Two models were tested. The first model was the original ESPRINT-15 model that included 4 factors, and the second model included the 2 factors we obtained from the EFA. Fig. 2 shows the path diagram for the original ESPRINT-15 model and the correlations between observed and latent variables. The path diagram for the second model is included in Supplement 4. MLR was used to calculate the correlations between latent variables. Table 6 presents a summary of the model fit indices of ESPRINT-15. In model 1, the overall goodness-of-fit Chi-square was significant for the model (χ^2^ 201.78, df 71, p < 0.000) with an RMSEA value of 0,09. These results are indicative of acceptable model fit. Likewise, model 1 obtained the lowest SRMR values and a CFI value larger than superior to 0.95 compared to model 2. These findings supported the satisfactory fit of this model.

**Table 6:** Confirmatory factor analysis estimates of the ESPRINT-15 (n = 235).

	Model 1 (original ESPRINT-15 model)	Model 2 (new model from EFA)
χ^2^	201.78	488.82
df	71	76
p	p < 0.00	p < 0.00
CFI	0.96	0.86
TLI	0.95	0.84
RMSEA (90% CI)	0.09 (0.07–0.10)	0.15 (0.14–0.17)
SRMR	0.11	0.15

CFI comparative fit index, EFA: exploratory factor analysis, TLI Tucker-Lewis index, RMSEA root-mean square error of approximation, SRMR standardized root-mean square residual

These results were consistent with prior EFA and CFA and supported the original 4 domains model structure.

#### Concurrent criteria validity

Concurrent criteria validity was assessed through ESPRINT-15 and the overall score (item 15 of the instrument) correlation. These results are shown in Table 7. The correlations ranged between 0.21 and 0.49. The best correlation coefficient was found between the item 14 and the overall score (ρ: 0.49; 95% CI 0.38; 0.59).

**Table 7:** ESPRINT-15 correlations with overall score (item 15).

Correlation values	Spearman's rho (ρ)	Significance	95% CI	
			Lower	Upper
Item 1	0.41	<0.00	0.29	0.51
Item 2	0.21	0.00	0.08	0.33
Item 3	0.34	<0.00	0.21	0.45
Item 4	0.26	<0.00	0.13	0.38
Item 5	0.35	<0.00	0.23	0.46
Item 6	0.38	<0.00	0.25	0.48
Item 7	0.40	<0.00	0.28	0.50
Item 8	0.35	<0.00	0.23	0.46
Item 9	0.35	<0.00	0.23	0.47
Item 10	0.40	<0.00	0.28	0.51
Item 11	0.41	<0.00	0.30	0.52
Item 12	0.40	<0.00	0.28	0.51
Item 13	0.40	<0.00	0.28	0.50
Item 14	0.49	<0.00	0.38	0.59
Domain 1	0.39	<0.00	0.27	0.50
Domain 2	0.41	<0.00	0.29	0.52
Domain 3	0.43	<0.00	0.32	0.53
Domain 4	0.46	<0.00	0.35	0.56

CI: confidence interval

#### Discriminatory ability

We conducted a Kruskal-Wallis test to explore the discriminatory capacity of ESPRINT-15 between severities of the disease. We did not find statistical differences in all domains among patients with different levels of AR severity. The median values and p values for each domain were as follows: for the “sleeping” domain a median value of 2.80 (p = 0.28), for the “symptoms” domain a median value of 2.00 (p = 0.16), for the “psychological affectation” domain a median value of 1.66 (p = 0.66), and for the “daily activities” domain a median value of 1.66 (p = 0.41). In terms of the asthma history, there were no statistical differences among the two groups in the domain scores (Mann-Whitney *U* test; p > 0.005). The median of the ESPRINT-15 domains was similar in patients with asthma compared to patients without asthma (Supplement 5).

## Discussion

Cross-cultural adaptation of questionnaires for measuring HRQL in a new country, culture, and/or language requires that there is equivalence between the original questionnaire and the new version.^13^ The ESPRINT-15 questionnaire is considered suitable for use in a variety of contexts, including clinical practice, observational studies, and clinical trials to assess HRQL in adult patients with AR.^39^ The aim of this study was to cross-culturally adapt the ESPRINT-15 questionnaire to be used in Colombia in patients with AR. To date, no specific HRQL questionnaire has been adapted and psychometrically validated in Colombian adults with AR. The present study examined the comprehensibility, acceptability, feasibility, reliability, and validity of ESPRINT-15 in Colombia applying 2 methodological theories. The results suggest that ESPRINT-15 has adequate factorial variance, construct validity, internal consistency, and test-retest reliability to be implemented in Colombian patients with AR.

Overall, the cross-cultural adaptation of ESPRINT-15 was easy except for minor comprehensibility issues between the ESPRINT-15 Spain's questionnaire version and Colombian's version regarding items 3, 4, and 7. We observed comprehensibility problems because respondents did not understand some words that are culturally used in Spain (eg, "picor"). Most ESPRINT-15 items offer 2 alternative options to comprehend each question. Both alternative options are separated by the word “or” (“o” in Spanish) (eg, "el picor en la nariz o estornudos repetidos"*,* *"*el picor de los ojos o tener que rascarse los ojos"). Therefore, whether respondents did not understand a specific word, they have an alternative to understand the item. We did not modify the original ESPRINT-15 items because the issues reported by the responders did not compromise comprehensibility of any ESPRINT-15 item. The response rate was 100% suggesting acceptability of the instrument. Questionnaire's feasibility was similar to the values reported in the development of ESPRINT questionnaire with 7.1 min of time to administer.^39^

Internal consistency, to measure the homogeneity of items on a test, was assessed using the Cronbach's alpha coefficient and the McDonald's omega coefficients. The Cronbach's alpha value and the McDonald's value for the Colombian version of the ESPRINT-15 was 0.95. The overall alpha values for the items of the questionnaire ranged from 0.94 to 0.95, suggesting that ESPRINT15 has a coherent construct to be applied in clinical setting.

Values of McDonalds's Omega coefficient are considered a more robust measurement of internal consistency than values from Cronbach's Alpha as it corrects the underestimation bias of Cronbach's Alpha when the tau-equivalence assumption is disrupted.^51^ We obtained an overall McDonalds's Omega value of 0.95 suggesting a high correlation between single items and the overall score item of the Colombian version of the ESPRINT-15.

The calculated ICC for assessing reliability were adequate in all domains suggesting good test-retest reliability (ICC from 0.70 to 0.76). The internal consistency values were similar to those reported in the original Spanish version for Cronbach's alpha from 0.69 to 0.90, as well as for test re-test reliability with ICC values from 0.63 to 0.83.^52^ The small variations in the internal consistency values and reproducibility across both studies can be the consequence of different aspects such as the population sampled, the methods of assessments and the intervals between assessments. According to our findings, ESPRINT-15 is a reliable questionnaire to measure health status.

G-theory is a comprehensive framework that opened the perspectives of the measurement theories as it allows to estimate score consistency with reference to multiple sources of measurement error.^53^ However, its application in measurement studies is not popular. Some reasons for its low applicability can its use of technical vocabulary, an overlooked link with CCT, and problems finding a software for doing a G-theory analysis.^53^ In our study the G-theory reported that 68.2% of the variance was explained for the patients, comparable to the variance obtained in the CTT analysis. This finding reflects that AR patients are the primary source of variability in the score observed when the ESPRINT-15 was applied. It also suggests that there is a substantial variation in how patients self-appraise the effect of the AR disease in their life. We also found that time had an interaction with patients (0.30 of variance component, 19.82% of variance), likely due to the fact that allergy symptoms change over time. Although both measurement theories provided similar variance values, G-theory provides comprehensive multiple sources of variance, including interactions, at the same time.^30^ G-Theory estimates how specific changes in a measurement procedure might impact score consistency.^53^

We performed factor analysis to assess the internal structure of ESPRINT-15 when it was applied to Colombian adults with AR. In the EFA, according to the eigenvalues and scree plot, we identified a new model with 2 factors extracted. We labeled the domains psychological and physical domains based on the components both grouped. This new model suggested modifications in the original domains of ESPRINT-15 moving from 4 original domains to 2 domains. Thus, we conducted a CFA to compare the goodness of fit of the original model and the new model using several statistical tests. According to this analysis, the original model showed better parsimony, stability, and adequacy values than the new model. Thus, it was not necessary to make modifications to the original items. Our findings confirmed the original four domain structure of ESPRINT-15.^39^

We evaluated the concurrent validity of ESPRINT-15 measuring correlations between each item and the overall score (item 15). Correlations were positive but low. This finding is explained because the measurement scale for retrieving the score is different from the other 14 items.

Discriminatory ability was addressed using the Kruskal-Wallis test and Mann Whitney *U* test. No differences were found between domain values and degrees of severity of the disease. This finding can be explained because the sample size in patients with mild intermittent, moderate/severe intermittent and mild persistent strata was low. Previous studies reported a good discriminatory capacity of ESPRINT-15 in adults' patients with AR,^54^^,^^55^ and after 4 weeks of treatment with rupatadine.^5^ Finally, the Mann Whitney *U* test did not show differences between patients with AR and with and without history of asthma. This result is similar to a previous finding reported in the process of developing the ESPRINT questionnaire^39^ and confirms that ESPRINT-15 was designed for assessing HRQL focused on AR symptoms but not symptoms related to asthma.

In conclusion, and according to our findings, ESPRINT-15 can be used as a routine clinical tool to assess HRQL outcomes in Colombian adult patients with AR. The use of this questionnaire in clinical practice would help to understand the impact of AR on quality of life and would improve its management with a patient-centric approach.

### Strengths and limitations

The strengths of our study are various. First, our sample size for the second stage allowed us to obtain reliable statistic results. Similarly, we addressed the statistical models via EFA and CFA with rigorous statistical methods for extraction and interpretation of data. Second, we found that ESPRINT-15 produced highly reliable scores, suggesting that the questionnaire can discriminate among patients with different AR-HRQL states. Third, our reliability findings using CTT, and G-theories were consistent between both techniques suggesting that these frameworks are useful for conducting cross-cultural adaptation studies, especially in chronic respiratory conditions such as AR. Fourth, we found that an interaction between time and AR patients accounted for significant variability among patients. This finding shows that ESPRINT-15 can be useful for clinicians as it will provide additional documentation regarding the effectiveness of treatments. There are some limitations in our study. First, we conducted one pilot test and it included a relatively small sample size. Further studies using adequate samples and multiple pilot tests for assessing comprehensibility, acceptability, and feasibility would provide useful additional evidence for the measurement performance of ESPRINT-15. Second, most patients in this study reported moderate/severe AR health status; thus, our findings may not be generalized in the context of patients with mild states.

### Future directions

It is possible that ESPRINT-15 questionnaire requires additional adjustments such as simplifying its findings in 2 factors or reducing the number of items to improve its routine applicability in the clinical context. Likewise, this cross-cultural adaptation will allow us to start adapting other HRQL questionnaires to Colombia as well as in other Spanish speaking countries using CTT or G-theories frameworks. In addition, further studies should be implemented to obtain population reference values and the Minimal Clinically Important Difference (MCID), which will aid to improve its interpretability.

## Conclusion

ESPRINT-15 was successfully cross-culturally adapted using both CTT and G-theory. Therefore, the ESPRINT-15 questionnaire is suitable for measuring HRQL in Colombian adult patients with AR. The construct validity, internal consistency and test re-test reliability were adequate obtaining an equivalent questionnaire to the original version to be applied in clinical contexts and epidemiological studies in Colombian adult patients with AR. G-theory that are useful for assessing reliability measures in cross-cultural adaptation studies.

## Abbreviations

AR, allergic rhinitis; ARIA, Allergic Rhinitis and its Impact on Asthma; CFA, confirmatory factor analysis; CFI, confirmatory factor index; CTT, classic test theory; EFA, exploratory factor analysis; ESPRINT-15, Cuestionario ESPañol de Calidad de Vida en RINiTis; G-coefficients, generalizability coefficients; G-theory; generalizability theory; HRQL, health related quality of life; ICC, intra-class correlations; MCID, Minimal Clinically Important Difference; RMSEA, root mean square error of approximation; SD, standard deviation; SRMR, Standardized root mean square residual, TLI, Tucker–Lewis's index.

## Funding

All authors acknowledge financial provided by the Vice Presidency for Research & Creation publication fund at 10.13039/501100006070Universidad de los Andes.

## Author's contributions

Conceptualization: Juan José Yepes-Nuñez, Edison Morales Cardenas, Carolina Gómez-García, Lina Marcela Martínez, Madelen Manco Sepúlveda, Antonio Valero; Methodology: Juan José Yepes-Nuñez, Antonio Valero, Meghan M. McConnell; Formal analysis and investigation: Juan José Yepes-Nuñez, Meghan M. McConnell; Writing - original draft preparation: Juan José Yepes-Nuñez; Writing - review and editing: Juan José Yepes-Nuñez, Edison Morales Cardenas, Carolina Gómez-García, Antonio Valero, Meghan M. McConnell; Funding acquisition: Juan José Yepes-Nuñez, Edison Morales Cardenas; Resources: Juan José Yepes-Nuñez, Edison Morales Cardenas; Supervision: Juan José Yepes-Nuñez, Meghan M. McConnell.

## Ethics approval and consent to participate

All procedures performed in this study were in accordance with the ethical standards of 1964 Helsinki declaration and its later amendments or comparable ethical standards. Informed consent was obtained from all individual participants included in the study.

## Submission declaration

The contents of this paper have not been published previously and are not under consideration for publication in any other format.

## Availability of data and materials

The datasets used and/or analyzed during the current study are available from the corresponding author on reasonable request.

## Authors' consent for publication

All authors have seen and approved the submission of this version.

## Declaration of competing interest

The authors report no conflicts of interest. The authors alone are responsible for the content and writing of the paper.
