# Supplementary material for: Classic test and generalizability theories are both useful for cross-cultural adaptation of an allergic rhinitis health-related quality of life questionnaire
Source: World Allergy Organ J. 2021 Nov 29;14(12):100612. doi: 10.1016/j.waojou.2021.100612 (PMC8640560; doi:10.1016/j.waojou.2021.100612)
Supplement: Multimedia component 1 [file mmc1.docx]

**SUPPLEMENTS**

| **Supplement 1. Original English version of the original items of ESPRINT-15 (included scores for each item)** | | | | | | | | |
| --- | --- | --- | --- | --- | --- | --- | --- | --- |
| **During the last two weeks, how much have you been bothered by the following symptoms?** | | | | | | | | |
|  | **Symptoms** | **Not at all** | **Almost not at all** | **A little** | **Moderately** | **A bit** | **A lot** | **Very much** |
| 1 | Feeling of blocked or stuffed up nose | 0 | 1 | 2 | 3 | 4 | 5 | 6 |
| 2 | Liquid nasal mucous or water like | 0 | 1 | 2 | 3 | 4 | 5 | 6 |
| 3 | Itchy nose or repeated sneezing | 0 | 1 | 2 | 3 | 4 | 5 | 6 |
| 4 | Itchy eyes or having to rub eyes | 0 | 1 | 2 | 3 | 4 | 5 | 6 |
| 5 | Difficulty in breathing or feeling of suffocation or shortness of breath | 0 | 1 | 2 | 3 | 4 | 5 | 6 |
| **During the last two weeks, how much have you been bothered by each one of the following symptoms?** | | | | | | | | |
|  | **Daily activities** | **Not at all** | **Almost not at all** | **A little** | **Moderately** | **A bit** | **A lot** | **Very much** |
| 6 | Discomfort or difficulty while working caused by your rhinitis | 0 | 1 | 2 | 3 | 4 | 5 | 6 |
| 7 | Rhinitis symptoms having dinner or while eating out | 0 | 1 | 2 | 3 | 4 | 5 | 6 |
| 8 | Constant interruption of what you are doing caused by your rhinitis | 0 | 1 | 2 | 3 | 4 | 5 | 6 |
| **During the last two weeks, how much have you been bothered by each one of the following symptoms?** | | | | | | | | |
|  | **Sleeping** | **Not at all** | **Almost not at all** | **A little** | **Moderately** | **A bit** | **A lot** | **Very much** |
| 9 | Problems getting to sleep or sleeping caused by your rhinitis. | 0 | 1 | 2 | 3 | 4 | 5 | 6 |
| 10 | Getting up dry mouthed or waking up because of it, caused by your rhinitis | 0 | 1 | 2 | 3 | 4 | 5 | 6 |
| 11 | Sleep badly, caused by rhinitis | 0 | 1 | 2 | 3 | 4 | 5 | 6 |
| **During the last two weeks, how much have you been bothered by each one of the following symptoms?** | | | | | | | | |
|  | **Psychological affectation** | **Not at all** | **Almost not at all** | **A little** | **Moderately** | **A bit** | **A lot** | **Very much** |
| 12 | Having to be on top of your rhinitis | 0 | 1 | 2 | 3 | 4 | 5 | 6 |
| 13 | Being more irritable or in a bad mood because of your rhinitis | 0 | 1 | 2 | 3 | 4 | 5 | 6 |
| 14 | Feel bad or have a bad time of it because of your rhinitis | 0 | 1 | 2 | 3 | 4 | 5 | 6 |
| **During the last two weeks, how much have you been bothered by each one of the following symptoms?** | | | | | | | | |
|  | **General health** | | | | | | | |
| 15 | In general, and only taking into account your rhinitis, how would you say your health is? | | | | | | | |
|  | Excellent Very good Good Normal Bad | | | | | | | |
| *This is not the official English version of the ESPRINT-15 questionnaire | | | | | | | | |

| **Supplement 2. Descriptive results of the pilot comprehension test of the ESPRINT-15** | | | |
| --- | --- | --- | --- |
| **English version of the ESPRINT-15 questionnaire*** | **Item understanding** | | **Decision** |
| **During the last two weeks, how much have you been bothered by the following symptoms?** | | | |
| **Symptoms** | | | |
| 1. Feeling of blocked or stuffed up nose. | Yes  No | 32 (100%)  0 (0%) | No changes were made |
| 2. Liquid nasal mucous or water like | Yes  No | 32 (100%)  0 (0%) | No changes were made |
| 3. Itchy nose or repeated sneezing | Yes  No | 31 (96.8%)  1 (3.2%) | No changes were made |
| 4. Itchy eyes or having to rub eyes | Yes  No | 31 (96.8%)  1 (3.2%) | No changes were made |
| 5. Difficulty in breathing or feeling of suffocation or shortness of breath | Yes  No | 32 (100%)  0 (0%) | No changes were made |
| **During the last two weeks, how much have you been bothered by each one of the following symptoms?** | | | |
| **Daily activities** | | | |
| 6. Discomfort or difficulty while working caused by your rhinitis | Yes  No | 32 (100%)  0 (0%) | No changes were made |
| 7. Rhinitis symptoms having dinner or while eating out | Yes  No | 31 (96.8%)  1 (3.2%) | No changes were made |
| 8. Constant interruption of what you are doing caused by your rhinitis | Yes  No | 32 (100%)  0 (0%) | No changes were made |
| **During the last two weeks, how much have you been bothered by each one of the following symptoms?** | | | |
| **Sleeping** | | | |
| 9. Problems getting to sleep or sleeping caused by your rhinitis. | Yes  No | 32 (100%)  0 (0%) | No changes were made |
| 10. Getting up dry mouthed or waking up because of it, caused by your rhinitis | Yes  No | 32 (100%)  0 (0%) | No changes were made |
| 11. Sleep badly, caused by rhinitis | Yes  No | 32 (100%)  0 (0%) | No changes were made |
| **During the last two weeks, how much have you been bothered by each one of the following symptoms?** | | | |
| **Psychological affectation** | | | |
| 12. Having to be on top of your rhinitis | Yes  No | 32 (100%)  0 (0%) | No changes were made |
| 13. Being more irritable or in a bad mood because of your rhinitis | Yes  No | 32 (100%)  0 (0%) | No changes were made |
| 14. Feel bad or have a bad time of it because of your rhinitis | Yes  No | 32 (100%)  0 (0%) | No changes were made |
| **During the last two weeks, how much have you been bothered by each one of the following symptoms?** | | | |
| **General health** | | | |
| 15. In general and only taking into account your rhinitis, how would you say your health is? | Yes  No | 32 (100%)  0 (0%) | No changes were made |
| *This is not the official English version of the ESPRINT-15 questionnaire | | | |

**Supplement 3. Scree plot exploratory factor analysis**


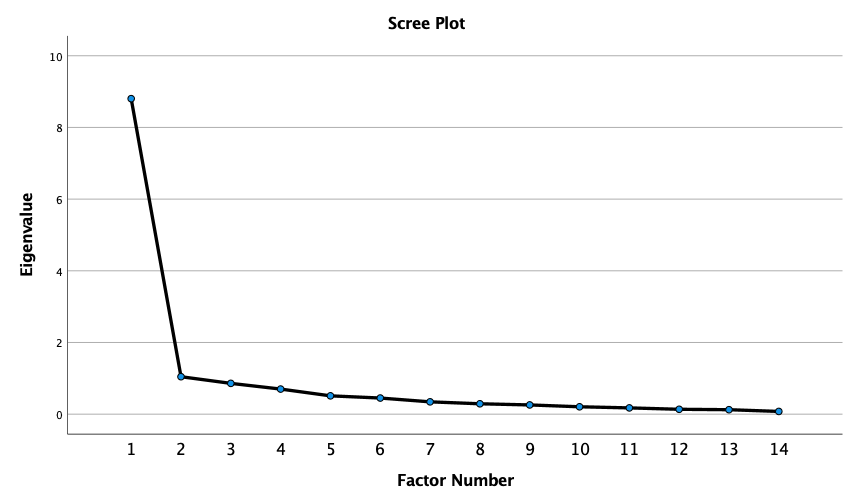


**Supplement 4. ESPRINT-15 two factors model with correlations between latent factors and observed items**


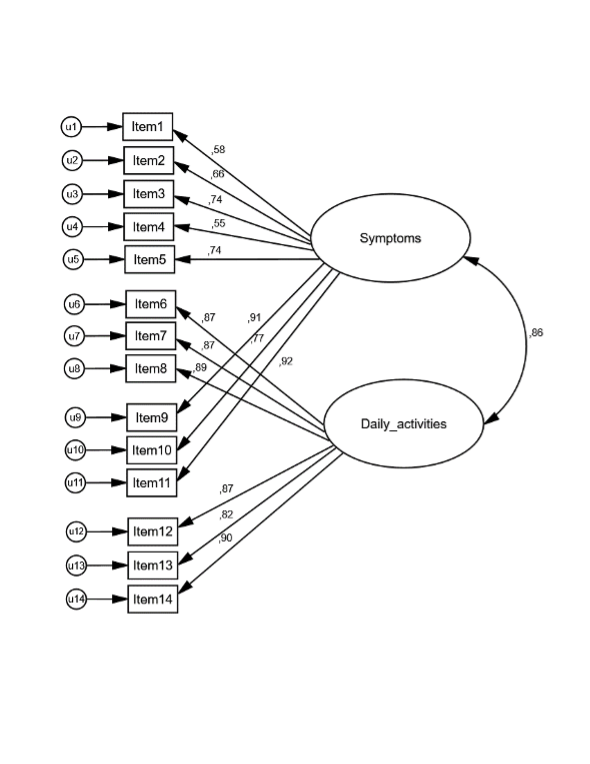


| **Supplement 5. Comparison of ESPRINT-15 domain scores between AR patients with asthma and without asthma** | | | | |
| --- | --- | --- | --- | --- |
| **ESPRINT-15 domains** | **AR patients with asthma**  **Median (IQR)** | **AR patients without asthma**  **Median (IQR)** | **Mann –Whitney**  **U test** | **P-value** |
| **Sleeping** | 3.00 (2.00 – 4.00) | 2.50 (1.80 – 3.80) | 5991.50 | 0.18 |
| **Symptoms** | 2.00 (1.00 – 3.00) | 2.00 (0.66 – 3.00) | 6209.50 | 0.37 |
| **Psychological affectation** | 2.00 (0.66 – 3.33) | 1.66 (0.66 – 3.00) | 6679 | 0.98 |
| **Daily activities** | 2.00 (0.33 – 3.25) | 1.66 (0.33 – 3.33) | 6464 | 0.69 |
| IQR: Interquartile ranges | | | | |
